# Supplementary material for: Neuromuscular electrical stimulation for physical function maintenance during hematopoietic stem cell transplantation: Study protocol
Source: PLoS One. 2024 May 10;19(5):e0302970. doi: 10.1371/journal.pone.0302970 (PMC11086915; doi:10.1371/journal.pone.0302970)
Supplement: S2 File — (PDF) [file pone.0302970.s002.pdf]

Human Subjects Protocol

VA Puget Sound IRB

Neuromuscular electrical stimulation for physical function maintenance during hematopoietic stem cell transplantation

MIRB 01879

Funding Agency: VA (RR&D)

Principal Investigator: Lindsey Anderson, PhD

09/14/2023

Abstract

Hematopoietic cell transplantation (HCT) is often indicated for the treatment of hematologic malignancies, but often leads to deconditioning, fatigue, muscle atrophy/ sarcopenia, and poor overall quality of life (QOL). However, there are no approved treatments available to prevent/reverse deconditioning, fatigue, or sarcopenia in the cancer setting. Various disease- and treatment-related factors lead to greatly reduced ability to exercise and participate in physical therapy, creating the need for alternative exercise strategies. Neuromuscular electrical stimulation (NMES) improves leg muscle strength and mass in non-cancer patient cohorts. The use of NMES to combat disuse atrophy and functional decline may be particularly useful in the HCT setting as patients undergo intensive preparatory chemotherapy and often experience symptoms including severe fatigue that leave them inactive or isolated for extended time periods surrounding the transplant.

The overall goals of this study are to assess 1) the efficacy of an NMES vs Sham intervention on HCT-induced reductions in physical function and muscle mass and worsening of patient-reported fatigue and QOL and 2) the association between physical function and prolonged recovery of patient-reported fatigue and QOL. We hypothesize that 1) NMES will attenuate the acute HCT-induced negative impact on physical function, body composition, QOL, and fatigue compared to Sham intervention, and 2) baseline physical function will be a significant predictor of 6-month recovery of physical function, patient-reported fatigue, and QOL. This will be a randomized (1:1, NMES:Sham; N=23/ group; 46 total subjects), and stratified by diagnosis. The subjects and the PI will be blinded to group assignment. One study coordinator will be unblinded in order to train each subject on use of NMES or Sham device. A different blinded coordinator will conduct study visit testing to avoid bias.

Physical function, body composition, QOL, and fatigue will be assessed at baseline (“Pre” after admission to the Bone Marrow Transplant Unit but before initiation of preparatory chemotherapy), 24±5 days after HCT (Follow-up 1; “FU1”), and 6-months after HCT (Follow-up 2; “FU2”). The primary outcome will be between-group difference in 6-Minute Walk Test change at FU1. Secondary outcomes include: body composition measured by dual-energy x-ray absorptiometry; previously validated questionnaires; standard of care clinical/laboratory data; circulating markers of fatigue; and NMES process measures such as feasibility, acceptability, adherence, accurate use, duration/intensity, complications, and satisfaction. Each patient will begin the intervention after the Baseline visit, completing three sessions/week, and continuing until three weeks after HCT. Each training day will be one-hour total: the first 30 minutes will consist of simultaneous bilateral NMES/Sham of the gluteals and hamstrings, followed by 30 minutes of bilateral NMES/Sham of the quadriceps.

List of Abbreviations

6MWT	6-Minute Walk Test
aLBM	appendicular lean body mass
ALT	alanine transaminase
ANOVA	analysis of variance
ASAS	Anderson Symptom Assessment Scale
AST	aspartate transaminase
BIA	bioelectrical impedance analysis
DXA	dual-energy x-ray absorptiometry
FACIT-F	Functional Assessment of Chronic Illness Therapy-Fatigue
FU	follow-up
HCT	hematopoietic stem cell transplantation
HGS	handgrip strength
LBM	lean body mass
MFI-20	Multidimensional Fatigue Inventory
MTU	marrow transplant unit
NMES	neuromuscular electrical stimulation
POMS	Profile of Mood States
PRO	patient-reported outcome(s)
QOL	quality of life
S/AE	serious/adverse event(s)
SCP	stair climb power
SF-36	Short Form-36 Health Survey
STS	five-times-sit-to-stand
VO_2peak	peak aerobic (oxygen volume) capacity

Contents

Protocol Title: 5
1.0 Study Personnel 5
2.0 Introduction 5
3.0 Objectives 6
4.0 Resources and Personnel 7
5.0 Study Procedures 8
5.1 Study Design 8
5.2 Recruitment Methods 11
5.3 Informed Consent Procedures 11
5.4 Inclusion/Exclusion Criteria 11
5.5 Study Evaluations 12
5.6 Data Analysis 15
5.7 Withdrawal of Subjects 17
6.0 Reporting 17
7.0 Privacy and Confidentiality 18
8.0 Communication Plan 19
9.0 Information Security and Data Storage/Movement 19
10.0 References 20

Protocol Title: Neuromuscular electrical stimulation for physical function maintenance during hematopoietic stem cell transplantation

1.0 Study Personnel

Study Team Member	VA Status	Degrees	Study Role	
Lindsey Anderson	8/8	PhD	PI	
Jose Garcia	8/8	MD, PhD	Co-Investigator	
Solomon Graf	8/8	MD	Co-Investigator	
Thomas Chauncey	8/8	MD, PhD	Co-Investigator	
Gary Miranda	WOC	LPN	Coordinator	
Lauren Paulsen	WOC	BS	Lead Coordinator	

2.0 Introduction

Over 60,000 hematopoietic stem cell transplants (HCT) are performed worldwide each year as a treatment for hematologic malignancies [1]. Moreover, upwards of 3,500 Veterans are diagnosed with a hematologic malignancy each year within the Veterans Affairs system [2]. This complex treatment involves exposure to extremely high doses of chemotherapy and radiation to eliminate cancerous/abnormally proliferating cells in the body in preparation for the HCT, leaving the immune system ablated. After HCT, the immune system rebuilds from donor (allogenic) or patient-derived (autologous) stem cells that are harvested and treated before implantation during HCT. However, HCT often leads to long-term deconditioning, fatigue, muscle atrophy/sarcopenia, and poor

overall quality of life (QOL) [3], which are often associated with complications such as hospitalization and infection [4]. The cancer itself, side effects of chemotherapy/ radiation, drug treatment, and other factors such as sedentary behavior likely contribute to this clinical presentation [5]. These disease- and treatment-related factors lead to greatly reduced ability to exercise and participate in physical therapy, which contribute to functional decline throughout the HCT process. In addition, patients with more functional impairment respond worse, and are offered less treatments due their poor functional status. Despite the significance of these symptoms, there are no approved treatments available to prevent/reverse deconditioning, fatigue, or muscle atrophy/sarcopenia in the cancer setting.

This study will examine the impact of a rehabilitative therapeutic modality, NMES, administered during HCT on physical function, fatigue, and QOL. The use of NMES to combat functional impairment and fatigue may be particularly useful in the HCT setting as it may be safely used daily without requiring supervision or for the patient to leave the treatment floor. The information derived from this study will be used for development of future rehabilitative strategies to maintain/restore physical function during the HCT process. HCT provides an optimal test of an NMES intervention since this acute cancer treatment is relatively more severe, more standardized, and shorter in duration than most other cancer treatments, but often produces long-lasting detriments to physical function and QOL. This study will allow the advancement of knowledge about a currently underutilized therapeutic strategy aimed to improve rehabilitative patient care and QOL by maintaining daily function and reducing the impact of a potent and widely used cancer treatment. This study will also be the first to examine the impact of HCT, with or without NMES, on physical function and patient-reported outcomes (PRO) in Veterans. Furthermore, since only three VA centers perform HCT, these results will be easy to implement in a real clinical scenario.

3.0 Objectives

Aim 1: To determine the efficacy of NMES vs. Sham for attenuation of HCT-induced reductions in physical function, muscle mass, and patient-reported QOL and fatigue in patients undergoing autologous HCT. Patients will be randomized 1:1 (NMES:Sham) stratified by diagnosis. Physical function, body composition, QOL, and fatigue will be assessed at baseline (Pre, after admission to the Bone Marrow Transplant Unit but before initiation of preparatory chemotherapy) and 24±5 days after HCT (Follow-up 1; FU1). The **primary outcome** will be between-group difference in 6MWT change at FU1 compared to Pre (N=23/group; 46 total). Secondary outcomes include: body composition measured by dual-energy x-ray absorptiometry (DXA); previously validated questionnaires (Functional Assessment of Chronic Illness Therapy-Fatigue; Muscle and

Joint Measures) to assess patient-reported fatigue, QOL, symptom burden, and functional status; standard of care clinical/laboratory data regarding co-morbidities, adverse events, hospitalizations, treatment history, functional status, and clinical course; and NMES process measures such as feasibility, acceptability, adherence in number of sessions, accurate use, duration/intensity, complications, and satisfaction.

Aim 2: To determine predictive ability of baseline 6MWT on delayed recovery of physical function, QOL, and fatigue, patient-reported outcomes, physical function, and chart review will be collected 6-months after HCT (FU2). 6MWT at Pre will be used to determine significant predictors of QOL and fatigue at FU2 (N=46) as assessed by previously validated questionnaires. Clinical measures extracted from medical charts will include changes from FU1 to FU2 in standard of care clinical/laboratory data regarding co-morbidities, adverse events, hospitalizations, functional status, treatment history, disease trajectory, and survival. **Aim 3 (exploratory)**: To investigate the acute impact of NMES vs. Sham during HCT on various aspects of physical function and patient-reported QOL for determining potential endpoints for future clinical trials. Exploratory measures of function (stair climbing power; muscle strength; sit-to-stand; handgrip strength; peak oxygen consumption, balance test) assessed at Pre and FU1 will be used to determine significant predictors of exploratory QOL measures (Multidimensional Fatigue Inventory; Short Form-36; European Organization for Research and Treatment of Cancer QOL Questionnaire) assessed at FU2.

- We hypothesize that: 1) NMES will attenuate the acute HCT-induced negative impact on physical function, body composition, QOL, and fatigue compared to Sham intervention, and 2) baseline physical function will be a significant predictor of 6-month recovery of patient-reported fatigue and QOL.

4.0 Resources and Personnel

- This research will be conducted at the VAPSHCS BMTU by the team of investigators described above.
- Dr. Anderson will direct the study and have oversight responsibilities with the help of Drs. Garcia and Chauncey. Dr. Chauncey and Graf will also provide guidance to the study and be the oncology experts for consultation. Dr. Anderson, Ms. Paulsen, and Mr. Miranda will perform study procedures. All will have access to protected health information (PHI). Drs. Anderson, Garcia, Chauncey, and Graf and Ms. Paulsen and Mr. Miranda will be involved in recruiting subjects and obtaining informed consent. Dr. Anderson, Ms. Paulsen, and Mr. Miranda will be involved in administering survey/interview procedures; Drs. Anderson, Garcia, Chauncey, and Graf will be performing data analysis. Drs. Garcia, Chauncey, and Graf will be involved in supervising the aerobic capacity test, which requires physician supervision.

5.0 Study Procedures

5.1 Study Design

- The overall goal of this randomized, sham-controlled pilot study is to assess the efficacy of an NMES vs Sham intervention on standard of care HCT-induced reductions in physical function and muscle mass and worsening of patient-reported fatigue and QOL and the association between acute changes and prolonged recovery of physical function and QOL. Measurements of physical function, body composition, and QOL will be assessed at Pre (after enrollment in VAPSHCS MTU but before initiation of preparatory chemotherapy) and 24 ± 5 days after HCT (Follow-up 1; FU1). Physical function, PRO (including QOL and fatigue), and chart review will be assessed 6-months after HCT (FU2).
- While this study is not double-blinded, we are taking precautions to try to minimize the influence of tester bias. During the Informed Consent process, patients will be instructed to not presume group their assignment. After Informed Consent is received, the unblinded study coordinator will randomize each participant to NMES:Sham on a 1:1 basis, stratifying by diagnosis. The unblinded coordinator will ensure each participant performs the appropriate NMES or Sham stimulus on each training day; stimulation will only occur in private exam rooms. NMES and Sham stimulators will be outwardly identical apart from a concealed “A” or “B” label indicating NMES or Sham device; the designation of A or B will only be known to the unblinded study coordinator, and not to the PI. The PI will administer all Pre and FU1 assessments and conduct all statistical analyses; therefore, the PI will remain blinded to NMES vs Sham group assignment until study completion. In the study database, group assignment will be coded as A or B, but the PI will not know the coding of A or B in relation to NMES or Sham. A document containing the coding of A and B, will be kept in a different electronic location than the study folder.
- The Pre study visit will occur as soon as possible after patient enrollment into the MTU and obtaining informed consent in tandem with an independently scheduled face-to-face clinic visit, with completion of this visit occurring before initiation of preparatory chemotherapy. After the Pre visit, participants will be randomized as soon as possible (1:1) to NMES or Sham, stratified by diagnosis. The first session of the intervention will be entirely supervised by an unblinded study coordinator where participants receive written instruction on safety, proper manipulation of the muscle stimulator, placement of the electrodes, and will become acquainted with the stimulus sensation. In effort to reduce face-to-face interactions in consideration of COVID restrictions, all subsequent sessions will be unsupervised, and the patient can complete the intervention independently at home. On each intervention day, an unblinded study coordinator will check in with the patient over the phone to 1) assess for adverse events

(AE) occurring since the end of the prior session, including muscle soreness and lab abnormalities, 2) remind the patient about proper electrode placement before initiation of the current session, and to check for any changes in skin color, moisture, integrity and/or sensitivity, and 3) to ask about engagement in other physical activities for the purpose of exercise since the previous NMES session. Each patient will begin the intervention after the Baseline visit, completing three sessions/week, and continuing until three weeks after HCT. Each training day will be one hour total: the first 30 minutes will consist of simultaneous bilateral NMES/Sham of the gluteals and hamstrings, followed by 30 minutes of bilateral NMES/Sham of the quadriceps. Participants will not be exempt from consultation for, or participation in, regular physiotherapy treatment at the discretion of their treating oncologist/ hematologist. If a physiotherapy consult is initiated at any time between Pre and FU1, the physiotherapist will be informed of the patients' study participation and group assignment by an unblinded study coordinator; all physiotherapy exposure between Pre and FU2 will be recorded.

- NMES will be delivered with the RS-4i Plus Sequential Stimulator (RS Medical, Vancouver, WA; US Food and Drug Administration-approved 2012). Parameters for stimulation will include: asymmetric biphasic waveforms at 71 pulses per second frequency (Hz), 0-400 µs pulse duration, 5:10s on:off time (33% duty cycle), and 1.5s ramp-up time. Participants will be in control of the muscle stimulator devices at all times and will be instructed to perform all sessions in the supine position. Bilateral NMES will be delivered via asymmetric, biphasic using four cutaneous parallel channels delivered simultaneously using 2"x4" or 3"x5" self-adhesive electrodes. For the active NMES group, participants will be encouraged to increase the amplitude to a level of moderate discomfort, such as that experienced during conventional exercise, but not to induce pain. At minimum, the amplitude should induce visible muscle contraction. For the Sham group, the first 2 minutes will be the exact same as the active NMES parameters; however, the amplitude will ramp down to 0 milliamps over the third minute and will remain at 0 milliamps for the remaining 28 minutes of the session. Since response to NMES is variable, some patients may have low tolerance due to high sensitivity. The unblinded study coordinator will be trained in skeletal muscle motor point sensitivity and identification, and will in turn, preemptively instruct each participant how to adjust electrode placement away from motor points for comfort as necessary. Alternatively, for patients with elevated cutaneous sensitivity, larger electrodes may also be used to increase comfort level by dispersing the current over a larger surface area.
- This study will recruit 46 (N=23/group, active NMES or Sham group) Veterans enrolled at the VAPSHCS MTU for planned autologous HCT.
- All study staff are trained for appropriate collection of informed consent, including full explanation of all potential study risks. Study staff are

adequately trained on procedure risks for performing DXA scan, blood draw, NMES administration [(1) electrical surge or shock should the equipment malfunction; (2) skin irritation or allergy at the electrode sites; (3) pain during treatment if the current amplitude is not adjusted slowly and on the basis of patient feedback; and (4) post-treatment muscle soreness], and physical function testing. To minimize risk of NMES/Sham during the study intervention, before initiation of each training session, study staff will assess for 1) adverse events (AE) occurring since the end of the prior session (including muscle soreness), 2) changes in skin integrity and/or sensitivity, and 3) proper electrode placement. To minimize risk to patients from research procedures at study visits (Pre, FU1, FU2), participants will conduct all study visit procedures under the direct supervision of trained study staff who will continually ask for feedback from patients regarding the need for breaks and/or the experience of pain/discomfort. In addition, participants will be reminded of the voluntary nature of the research procedures, including the option to skip any procedure(s) they do not feel safe attempting or should not medically perform (i.e. VO_2peak will not be performed in patients with coronary artery disease), or question(s) that they do not feel comfortable answering. Other precautions include: 1) VO_2peak will always be supervised by a physician, 2) we will take sterile precautions by keeping the venipuncture site clean and dry, and 3) a snack will be available once fasting-required research procedures are completed. All VA sensitive research information will be maintained within the VA-secured network on password-protected computers (electronic data) and behind double-lock in VA-secured research laboratory/office space in Building 1, Room 815 (paper data).

- Subjects will be asked to sign a separate consent form for enrolling in the Geriatric Endocrine Repository for data and specimen storage.

5.2 Recruitment Methods

- 46 (N=23/group, active NMES or Sham group) subjects are needed
- When a patient is being evaluated for enrollment into the MTU, clinical staff at the MTU will introduce the study to the patient and ask if they are interested in learning more. If they agree, then research staff will be made aware that the patient is amenable to being approached at a clinical visit.
- Participants will receive a $50 compensation payment via Electronic Funds Transfer (EFT) 1) at the completion of Pre, 2) after completion of FU1, and 3) after study staff receive completed PRO questionnaires for FU2 for a total possible monetary compensation of $150.

5.3 Informed Consent Procedures

- A waiver of informed consent for pre-screening/recruitment has been requested.
- Research staff will approach the patient at a clinical visit after receiving notification from clinical staff that the patient is amenable to being approached about the study. Research staff will inform the subject about the full nature of the study, including participation details and all possible risks/benefits. Conversations with patients about study participation will be held in private rooms to ensure privacy and confidentiality. All patients will be assured of 1) the voluntary nature of participation and 2) that their clinical care will not be altered in any way due to their decision to participate or not.
- Any new study personnel that are added to the protocol will shadow an experienced staff person during at least 3 consenting sessions to be trained regarding human subjects protections requirements and how to obtain and document informed consent for this protocol. Subsequently, each new study personnel will obtain consent with supervision by more experienced personnel for at least consenting sessions before being allowed to consent independently.

5.4 Inclusion/Exclusion Criteria

- **Inclusion criteria**:
  - At least 18 years of age
  - Adequate cognitive and language ability to provide consent
  - Veteran enrolled in MTU at VAPSHCS for planned standard of care autologous HCT
- **Exclusion criteria**:
  - Active deep vein thrombosis or thrombophlebitis of the lower body
  - Untreated hemorrhagic disorders
  - Concomitant study inclusion in other nutritional or physical exercise interventional trials
  - Concomitant use of anabolic agents
  - Rhabdomyolysis or other muscle conditions where NMES is contraindicated
  - Implanted cardiac device
  - Baseline patient-reported muscle soreness of 5-6 on the soreness likert scale that is unrelated to recent physical exertion
  - History of prior hematologic stem cell transplant

- Probable or definitive liver cirrhosis
- Reduced renal clearance defined as Stage 4 chronic kidney disease (glomerular filtration rate <45 ml/min/1.73 m^2)

5.5 Study Evaluations

- Measurements of physical function at Pre and FU1 will include: 6MWT, SCP, HGS, gait speed, 1-RM (repetition maximum) muscle strength, STS, VO_2peak, balance test, and physical activity levels. Physical function assessments at FU2 will consist of 6MWT and physical activity levels. Body composition will be measured by dual-energy x-ray absorptiometry (DXA) and/or bioelectrical impedance analysis (BIA) at Pre and FU1. PROs will be measured via questionnaires to assess QOL, fatigue, symptom burden, and functional status at Pre, FU1, and FU2. Additional data collected will include clinical and laboratory data acquired per standard of care pertaining to comorbidities, HCT complications, hospitalizations, treatment history, and clinical course and process measures such as feasibility, acceptability, adherence in number of sessions, accurate use, duration/intensity, and satisfaction. Hemoglobin, which transports oxygen in the circulation and is measured per standard of care in HCT recipients, will also be obtained as a physiologic parameter that could impact fatigue and other outcomes. In consideration of COVID risk, and due to the nature of this population being immuno-compromised, all outcomes that are feasible to be performed in the MTU and Bld 1/Rm 112 (DXA) will be assessed in those locations instead of Bldg 1/5th floor Garcia research lab location. The only outcomes that cannot be assessed in the MTU are 1RM (strength will be assessed alternatively, and temporarily, by handheld dynamometry in the MTU as described below), VO_2 peak, (will not be assessed at this time), SCP (will not be assessed at this time), and the DXA scan (will be assessed in Bld 1/ Rm 112). This change in location is a temporary change in consideration of COVID risk.
- **Primary outcome: 6-minute walk test (6MWT):** Participants will walk as quickly as possible, without running, back and forth down an open hallway, across a distance of 20 meters. Participants may stop as needed, but the timer will not pause; the total distance walked will be recorded after six continuous minutes. At Pre and FU1, 6MWT will be monitored and manually recorded by study staff and by a validated mobile-phone based application which uses accelerometry data to report the total distance walked, step timing, gait symmetry, and walking changes over time [41, 42]. The mobile application called iWalkAssess, is commercially available for free to download on both android and iOS platforms. At FU2, 6MWT will only be measured by iWalkAssess mobile application. For this measurement at FU2, study staff will phone the subject to remind them that we will be sending them an activity monitor and questionnaires by mail. We will also send them a 6MWT instruction page (which will contain a place to

write their test result) and a 20-meter long strand of yarn so they can accurately perform this test at home. There will be no PHI entered into this mobile application and no data of any kind will be transmitted from the subjects' phone.

- **Patient-reported outcomes (PRO) for quality of life (QOL):** Anderson Symptom Assessment Scale (ASAS), Muscle and Joint Measure (MJM), Functional Assessment of Chronic Illness Therapy-Fatigue (FACIT-F), Short Form-36 Health Survey (SF-36), and the Multidimensional Fatigue Inventory (MFI-20). PRO including fatigue parameters are ASAS, FACIT-F, SF-36, and MFI-20.
- **Body composition:** Measured by dual energy fan-beam x-ray absorptiometry (DXA; Hologic Inc., Marlborough, MA) and BIA. DXA uses low-dose radiation (0.8 millirem for one whole-body scan) to determine whole body and segmental fat mass, bone mass, and fat-free mass. Participants will dress in a hospital gown and undergarments for this scan. aLBM will be recorded as the sum of fat-free mass in the four limbs. For patients with unilateral limb amputation, the fat-free mass of the contralateral/intact limb will be duplicated for the missing/partial limb. This procedure requires an overnight fast. BIA is a noninvasive method of estimating body composition based on the ability of tissues to conduct an electrical current. Based on this principle, lean tissue conducts electricity better than fat; therefore, the body composition analyzer/scale is able to calculate total body water, total body fat and total body lean mass.
- **Peak aerobic capacity (VO_2peak): (temporarily not assessed in consideration of COVID risk due to inability to perform this assessment in MTU)** (under direct supervision of a physician) participants will be instructed to put on a mask/mouth piece with a breathing valve to collect expired gases via indirect calorimetry. Participants will be instructed to use hand signals to notify researchers about the need to stop the test. Participants will then be asked to pedal an exercise cycle ergometer at progressively harder workloads. The test will continue until the participant becomes fatigued and decides to stop, or other symptoms prohibit further exercise. Participants will be asked to rate their perceived exertion during the test using the Borg Scale. After testing, participants will perform a cool-down exercise where they continue to cycle at a self-selected pace with zero resistance on the ergometer for 3-5 minutes. Heart rate will be monitored electronically throughout the warm-up, exercise, and cool-down portions of the test. This test requires an overnight fast.
- **Handgrip strength (HGS):** Participants will squeeze a handheld dynamometer (Jamar Hydraulic Dynamometer, J.A. Preston Corp., Clifton, NJ) as hard as possible for five seconds. Three attempts will be made on each hand when possible, with the highest measure per hand recorded and averaged for reporting mean HGS.

- **Stair climbing power (SCP): (temporarily not assessed in consideration of COVID risk due to inability to perform this assessment in MTU)** Participants will ascend a flight of stairs at the highest possible speed, according to their capabilities. The stairs will consist of 13 steps of 15.3 cm each, thus covering a total vertical distance of 1.99 m. An experimenter will measure the time employed to complete the test with a digital stopwatch. Anaerobic power (in Watts) will be calculated as follows:

  [body mass (kg) x 9.81 (m/s^2) x vertical distance (m)] / time (s)

  where 9.81 m/s^2 represents the acceleration of gravity. Two-three practice trials will be allowed so that the participant gains good control of the technique, with the shortest time to completion selected for data analysis.
- **The Five-times-sit-to-stand test (STS):** Participants will begin seated, then stand up and sit down five times as quickly as possible, maintaining their arms crossed on their chest. STS may be performed up to three times to ensure test familiarization and the best performance will be recorded for analysis.
- **Gait speed:** Participants will be asked to walk at their usual pace for 4 meters, from a standing start. The time employed to walk the 4 meters will be used to calculate gait speed in meters/second and recorded for analysis.
- **One-repetition maximum (temporarily not assessed in consideration of COVID risk due to inability to perform this assessment in MTU; isometric strength via dynamometry will proceed):** Participants will complete maximal strength testing of three major lower body muscle groups on pneumatic exercise equipment (Keiser Corp., Fresno, CA): quadriceps (bilateral knee extension), hamstrings (bilateral knee flexion), and gluteals (unilateral hip extension). Participants will be fitted to each exercise machine in accordance with proper body mechanics. Before initiating any exercise, participants will be instructed on the strength protocol, proper form, and breathing technique and will be given reminder cues for proper form and breathing as needed throughout each exercise. Strength testing will begin with assessment of range of motion performed without any resistance, this will also allow the participants to get familiar with the appropriate stop/start points of each exercise. Then, two warm-up sets will be performed with resistance (1: 7-8 repetitions at approximately 5/10 difficulty, 2: 4-5 repetitions at approximately 7/10 difficulty) to increase circulation and neural recruitment to the muscle group(s). Next, participants will begin the one-repetition maximal strength attempts, with one-minute of rest in between, and with the goal of achieving maximal effort within 3-5 attempts. Maximal strength will be recorded from the last successful attempt while maintaining proper form and range of motion. Maximal isometric strength of the knee extensors and flexors will be assessed using handheld dynamometry (MicroFET 2; Performance Health, Warrenville, IL)

where patients push or pull against a foam-padded hand-held force transducer to assess their maximum force output. Participants will undergo 1-2 practice/warm-up attempts and then attempt 2-3 maximal attempts, with one-minute of rest in between.

- **Balance Test**: Tests of standing balance include tandem, semi-tandem, and side-by-side stands. For each stand, study staff will demonstrate the task, then support each subject with one arm while they position their feet, ask if they are ready, then release the support and began timing. The timing will be stopped when the participant moves their feet or grasps the study staff for support, or when 10 seconds had elapsed. Each participant will begin with the semi-tandem stand, in which the heel of one foot is placed to the side of the first toe of the other foot, with the participant choosing which foot to place forward. Those unable to hold the semi-tandem position for 10 seconds will be evaluated with the feet in the side-by-side position. Those able to maintain the semi-tandem position for 10 seconds will be further evaluated with the feet in full tandem position, with the heel of one foot directly in front of the toes of the other foot.
- **Physical activity:** We will use validated triaxial accelerometry (Actical watch) to determine changes in daily spontaneous physical activity. Actical will be mailed to patients ahead of their visit, information will be recorded for 7 days, and then collected at their visit day (when they return to clinic or when they return it by mail). The devices will be worn by the subjects on the wrist and trained research coordinators will demonstrate its use and provide subjects with instructions in person as well as in writing. Data from returned monitors will be downloaded with companion software and analyzed for activity counts and daily steps.
- **NMES feasibility and feedback:** Participants will be asked to complete an acceptability and feasibility form at the end of FU1 In addition, the NMES device has an internal function where users are required to rate their pain before and after each NMES session. This information will also be collected as part of acceptability and adherence.
- **Safety parameters:** Venipuncture will be performed to measure muscle damage (creatine kinase, CK), nutritional markers (pre-albumin, albumin, total protein), renal function (creatinine), liver function (AST, ALT), and hematological parameters (leucocytes, thrombocytes, hematocrit, hemoglobin, erythrocytes) in plasma and serum. CK will be measured at each study visit and every two weeks after initiation of NMES/Sham. In the case of a Grade 3 or higher elevation in CK (described below), a urinalysis will be performed to check for proteinuria, and Dr. Chauncey and/or Dr. Graf will be notified of the results as soon as possible. Other safety parameters will include AE (National Cancer Institute Common Terminology Criteria for Adverse Events v5.0), satisfaction related to use of NMES/Sham, and survival.

- CK elevations will be graded according to National Cancer Institute Common Terminology Criteria for Adverse Events v5.0 guidelines and the VA Puget Sound blood lab Upper Limit of Normal "ULN" (195 U/L):
  - Grade 1 (1-2.5x ULN: 195-487 U/L)
  - Grade 2 (2.5-5x ULN: 488-975 U/L)
  - Grade 3 (5-10x ULN: 976-1950 U/L)
  - Grade 4 (>10x ULN: 1950+ U/L)
  - An SAE will occur when a Grade 4 CK value is observed with a urinalysis indicating proteinuria and a score of 5-6 on the soreness scale

- Criteria for withholding/restarting the intervention:
  - Intervention will be held in the case of Grade 3 CK elevation regardless of urinalysis or muscle symptoms. CK will then be measured every 48 hours and the intervention may begin again when CK drops below 5xULN. This precaution will be re-assessed after 5-10 patients have completed the intervention to determine the frequency of CK elevations and associated symptoms. If Grade 3 occurs frequently without urine or muscle symptoms, then the CK elevation criterion to hold the intervention may be raised per study physician recommendation.
  - Intervention will be held in the case of patient-reported soreness score of 5-6. The study coordinator will ask the subject about the time course of soreness development and for any symptoms of systemic muscle damage such as darker than normal urine color. This information will be relayed to the study physician(s) for their determination of any necessary clinical follow-up. At minimum, soreness will be measured every 24 hours and the intervention may begin again when soreness falls between 0-4.

- **Standard of care data:** chart review will be conducted to collect clinical and laboratory data acquired per standard of care pertaining to demographics, sex, height, weight, vitals, diagnosis, co-morbidities, adverse events, hospitalizations, treatment history/ updates, medications, functional status, edema, and clinical course.

5.6 Data Analysis

- The primary outcome will be between-group difference in 6MWT change from Pre to FU1, as measured by manual assessment. With a between-group difference in 6MWT change of -20.7 ± 16.0m, Type I error probability α=0.05, power=0.9, and 1:1 control:experimental participants, we will need to study 15 minimum participants per group. This is consistent with the recent finding from Bewarder and colleagues in which a combined group of autologous/allogenic HCT patients and hematologic cancer patients undergoing intensive chemotherapy without HCT displayed a mean reduction in 6MWT of 24m after NMES [35]. We conservatively estimate a

50% attrition rate, and therefore estimate an enrollment goal of 23 participants/group (46 total).

- All variables will be summarized descriptively using N, median, and standard error using the latest version of SPSS on the secure VA network. The study cohort will be described using baseline (Pre) data, both overall and comparing between study arms using t-tests for continuous variables and X^2 tests for categorical variables. While we anticipate that the randomization will ensure any potential confounding factors (i.e. diagnosis and sex) are equally distributed across groups, these bivariate tests will allow us to ascertain whether this assumption is upheld or whether there are differences to control for as potential confounders in the multivariate analyses. If necessary, appropriate transformations will be applied to the outcome changes to improve distribution toward normality. All testing of statistical significance will be 2-tailed, and a difference resulting in a p-value of ≤ 0.05 will be considered statistically significant.
- **Aim 1**: Intent-to-treat analyses will be conducted within-groups using paired-sample t-tests for continuous variables and X^2 for categorical variables with comparison of primary, secondary, and safety outcomes between Pre and FU1. Independent t-tests will be used for between-group comparisons of FU1-Pre change variables for primary, secondary, and safety outcomes. Sub-analyses will be conducted excluding patients who did not meet the minimum adherence standard. We will also conduct a sensitivity analyses on personal factors such as mood, aversion to learning new technology, presence of a caregiver, co-morbidities, symptom burden, and number of weekly clinical visits that may impact adherence.
- **Aim 2**: Multiple linear regression models to test whether baseline (Pre) 6MWT is a predictor of physical function or patient-reported fatigue (ASAS or MJM) at FU2. The model will control for group assignment, adherence (number of sessions), hemoglobin, number of blood transfusions, and age as well as any potential confounding variables found to be unbalanced between groups in bivariate testing described above. Intent-to-treat analyses will be conducted within-groups using paired-sample t-tests for continuous variables and X2 for categorical variables with comparison of safety outcomes between FU1 and FU2. Time x group interactions will be analyzed using two-way ANOVA for between-group comparison of FU2-FU1 change values for safety outcomes.
- **Aim 3 (exploratory)**: Separate multiple linear regression analyses will be performed to determine the association between each exploratory functional outcome (SCP, HGS, gait speed, 1-RM, STS, VO_2peak, balance) at Pre or FU1 and 6MWT or patient-reported fatigue at FU2 assessed by exploratory PRO (SF-36, MFI-20, FACIT-F). Each model will control for group assignment, adherence [(number of sessions), hemoglobin, number of blood transfusions, and age as well as any potential confounding variables found to be unbalanced between groups in bivariate testing described above.

5.7 Withdrawal of Subjects

- It is unlikely that participants will be withdrawn without their consent. This may happen if the sponsor or regulatory authorities decide to terminate the study. Participation is completely voluntary, and patients may withdraw from this study at any time without any negative consequences or penalty for study withdrawal. To withdraw, patients will be instructed to advise study staff directly by calling the number in the consent form. Patients may withdraw their consent and discontinue at any time with verbal or written notification to the investigators.

6.0 Reporting

- **Adverse event management**

  Any untoward or unfavorable event occurring following enrollment in the study and until conclusion of the study procedures will be considered an AE. A worsening of a pre-existing condition will be considered an AE as well. A pre-existing condition which occurs with a known temporal frequency and severity will not be considered an AE unless the pattern or severity has changed. A serious adverse event (SAE) is any AE that results in: death, permanent or significant disability, hospitalization or a prolongation of an existing hospitalization, a congenital anomaly/birth defect, or is otherwise medically alarming. All AE will be rated according to the National Cancer Institute Common Terminology Criteria for AE v5.0.

- **Relationship to study procedure:** All AE will be assessed by the Investigator to establish the presumed causal relationship with the study procedure considering: a) Temporal relationship, b) Pattern consistent with known effect of the intervention, and c) Presence of other potential etiologies. AE will be assigned one of the following causal relationships: a) Unrelated (The study procedure almost certainly did not cause the event); b) Probably not related (It is more likely that the event is due to another etiology than due to the study procedure), c) Possibly related (It is equally likely that the event is due to the study procedure as it is due to another etiology), d) Probably related (It is more likely that the event is due to the study procedure than due to another etiology), and e) Definitely related (the evidence is compelling that the study procedure caused the AE).

- **Monitoring adverse events:** Subjects will be monitored for the onset of AE throughout the course of the study. Any ongoing AE will be assessed at appropriate frequency to document the date and time of resolution of the event. All events will be followed to resolution. Certain AE (e.g., a cerebrovascular accident) will not be expected to resolve completely; in these cases, the date and

time will be recorded when the event reaches its new, stable equilibrium and any remaining residua of the event will be documented. All AE will be followed until resolution or until stable in cases where permanent sequelae are expected. All “unanticipated and related SAE” and “unanticipated and related problem involving risk to subjects or others (UPIRTSO)” will be reported to IRB with appropriate forms per IRB regulations (within 5 business days of the reporting individual becoming aware of the event), all other AEs will be reported to IRB annually. The HRPP director will be notified orally of all unanticipated related deaths immediately after the reporting individual becoming aware of the event. The device manufacturer, RS Medical, will also be notified of all unanticipated and related SAEs within 5 business days of the reporting individual becoming aware of the event via Azure RMS email; RS Medical will be responsible for reporting these SAEs to the FDA.

- A Data Monitoring Committee will conduct a blinded examination of the safety data to ensure safety of subjects. All study AEs will be documented and tabulated for DMC reporting. Unanticipated and related SAEs will be reported to the DMC via Azure RMS email within 5 business days of the investigators becoming aware of the event. In addition, the DMC will meet to review safety reports every 6 months. This review will take place either in person or by teleconference. Each safety report will include the enrollment status, the number of subjects recruited, screened, and randomized. In addition, it will incorporate the number of dropouts and the reason for the dropouts. The report will list all adverse events and all SAE that did not require expedited reporting since the previous reporting period. The summary of all adverse events will allow for a comprehensive overview or analysis of the event rates. All AE’s will be evaluated for severity and attribution. In order to protect participant’s confidentiality, the data provided to the DMC will be coded so that individual patients cannot be identified.

7.0 Privacy and Confidentiality

- Data collected in this VA research study, including identifiers, will be maintained for no more than 6 years by the VA facility. All study documents containing PHI will be kept in a locked cabinet inside a locked room (in Dr. Anderson/Garcia’s research office 1/815) or behind VA firewall on VA password-protected computers on Dr. Anderson/Garcia’s secured research drive. No data containing PHI will be accessible outside the study site. The room is kept locked with the door shut. No subject data is left out of the cabinets. Confidential information will be stored on servers managed and maintained by the VA-IT program.

  An adequate plan exists to protect health information identifiers from improper use and disclosure to minimize the risk of loss of confidentiality as recommended by VA guidelines. No social security numbers will be requested from subjects or

potential subjects via phone Adequate written assurances exist in order to ensure that the PHI will not be reused or disclosed to (shared with) any other person or entity, except as required by law, for authorized oversight of the research study, or for other research for which the use or disclosure of the PHI would be permitted under the Privacy Rule to minimize the risk of loss of confidentiality as recommended by VA guidelines.

The PI, VA collaborators, VA research staff, and VA research compliance monitors will have access to identifiable information. People who ensure quality from the institutions where the research is being done, federal, and other regulatory agencies will have access to all of the research data. No one else will have access to identifiable research data. An Accounting of Disclosure will be created and maintained for any disclosure of individually identifiable information outside the VA. The manual spreadsheet will include the date of the disclosure, nature or description of the individually identifiable information disclosed, purpose of each disclosure and the name and address of person or agency to which the disclosure was made.

8.0 Communication Plan

- This is a single center study. Full disclosure and complete description of all endpoints will be included in public presentations of data to avoid bias. Safety, including AE will be summarized descriptively by treatment group. VA and funding sources will be acknowledged in all disclosures.

9.0 Information Security and Data Storage/Movement

- Data will be obtained from chart review or generated at each study visit. This data will be maintained for no more than 6 years by the VA facility. All study documents containing PHI will be kept in a locked cabinet inside a locked room (in Dr. Anderson/Garcia’s research office 1/815) or behind VA firewall on VA password-protected computers on Dr. Anderson/Garcia’s secured research drive. No data containing PHI will be accessible outside the study site. The room is kept locked with the door shut. No subject data is left out of the cabinets. Confidential information will be stored on servers managed and maintained by the VA-IT program.

10.0 References

1. Niederwieser, D., H. Baldomero, J. Szer, et al., Hematopoietic stem cell transplantation activity worldwide in 2012 and a SWOT analysis of the Worldwide Network for Blood and Marrow Transplantation Group including the global survey. Bone Marrow Transplant, 2016. **51**(6): p. 778-85.
2. Zullig, L.L., K.J. Sims, R. McNeil, et al., Cancer Incidence Among Patients of the U.S. Veterans Affairs Health Care System: 2010 Update. Mil Med, 2017. **182**(7): p. e1883-e1891.
3. Dirou, S., A. Chambellan, P. Chevallier, et al., Deconditioning, fatigue and impaired quality of life in long-term survivors after allogeneic hematopoietic stem cell transplantation. Bone Marrow Transplant, 2018. **53**(3): p. 281-290.
4. Kisch, A., S. Lenhoff, S. Zdravkovic, et al., Factors associated with changes in quality of life in patients undergoing allogeneic haematopoietic stem cell transplantation. Eur J Cancer Care (Engl), 2012. **21**(6): p. 735-46.
5. Copelan, E.A., Hematopoietic stem-cell transplantation. N Engl J Med, 2006. **354**(17): p. 1813-26.
6. Fukushima, T., J. Nakano, S. Ishii, et al., Characteristics of muscle function and the effect of cachexia in patients with haematological malignancy. Eur J Cancer Care (Engl), 2018: p. e12956.
7. Morishita, S., K. Kaida, T. Tanaka, et al., Prevalence of sarcopenia and relevance of body composition, physiological function, fatigue, and health-related quality of life in patients before allogeneic hematopoietic stem cell transplantation. Supportive Care in Cancer, 2012. **20**(12): p. 3161-3168.
8. Persoon, S., M.J. Kersten, L.M. Buffart, et al., Health-related physical fitness in patients with multiple myeloma or lymphoma recently treated with autologous stem cell transplantation. J Sci Med Sport, 2017. **20**(2): p. 116-122.
9. Hacker, E.D., C. Ferrans, E. Verlen, et al., Fatigue and physical activity in patients undergoing hematopoietic stem cell transplant. Oncol Nurs Forum, 2006. **33**(3): p. 614-624.
10. Hung, Y.C., J. Bauer, P. Horsley, et al., Changes in nutritional status, body composition, quality of life, and physical activity levels of cancer patients undergoing autologous peripheral blood stem cell transplantation. Supportive Care in Cancer, 2013. **21**(6): p. 1579-86.
11. Mitchell, S.A., N.K. Leidy, K.H. Mooney, et al., Determinants of functional performance in long-term survivors of allogeneic hematopoietic stem cell transplantation with chronic graftversus-host disease (cGVHD). Bone Marrow Transplant, 2010. **45**(4): p. 762-9.
12. Ware, J.E., Jr. and B. Gandek, Overview of the SF-36 Health Survey and the International Quality of Life Assessment (IQOLA) Project. J Clin Epidemiol, 1998. **51**(11): p. 903-12.

13. Liang, Y., M. Zhou, F. Wang, et al., Exercise for physical fitness, fatigue and quality of life of patients undergoing hematopoietic stem cell transplantation: a meta-analysis of randomized controlled trials. Jpn J Clin Oncol, 2018. **48**(12): p. 1046-1057.
14. Wiskemann, J., P. Dreger, R. Schwerdtfeger, et al., Effects of a partly self-administered exercise program before, during, and after allogeneic stem cell transplantation. Blood, 2011. **117**(9): p. 2604-13.
15. Hacker, E.D., E. Collins, C. Park, et al., Strength Training to Enhance Early Recovery after Hematopoietic Stem Cell Transplantation. Biol Blood Marrow Transplant, 2017. **23**(4): p. 659-669.
16. Jarden, M., K. Nelausen, D. Hovgaard, et al., The effect of a multimodal intervention on treatment-related symptoms in patients undergoing hematopoietic stem cell transplantation: a randomized controlled trial. J Pain Symptom Manage, 2009. **38**(2): p. 174-90.
17. Baumann, F.T., L. Kraut, K. Schule, et al., A controlled randomized study examining the effects of exercise therapy on patients undergoing haematopoietic stem cell transplantation. Bone Marrow Transplant, 2010. **45**(2): p. 355-62.
18. Laine, J., A. D'Souza, S. Siddiqui, et al., Rehabilitation referrals and outcomes in the early period after hematopoietic cell transplantation. Bone Marrow Transplant, 2015. **50**(10): p. 1352-7.
19. Picorelli, A.M., L.S. Pereira, D.S. Pereira, et al., Adherence to exercise programs for older people is influenced by program characteristics and personal factors: a systematic review. J Physiother, 2014. **60**(3): p. 151-6.
20. Adams, V., Electromyostimulation to fight atrophy and to build muscle: facts and numbers. J Cachexia Sarcopenia Muscle, 2018.
21. Di Filippo, E.S., R. Mancinelli, M. Marrone, et al., Neuromuscular electrical stimulation improves skeletal muscle regeneration through satellite cell fusion with myofibers in healthy elderly subjects. J Appl Physiol (1985), 2017. **123**(3): p. 501-512.
22. Kern, H., L. Barberi, S. Lofler, et al., Electrical stimulation counteracts muscle decline in seniors. Front Aging Neurosci, 2014. **6**: p. 189.
23. Zampieri, S., S. Mosole, S. Lofler, et al., Physical Exercise in Aging: Nine Weeks of Leg Press or Electrical Stimulation Training in 70 Years Old Sedentary Elderly People. Eur J Transl Myol, 2015. **25**(4): p. 237-42.
24. Jones, S., W.D. Man, W. Gao, et al., Neuromuscular electrical stimulation for muscle weakness in adults with advanced disease. Cochrane Database Syst Rev, 2016. **10**: p. CD009419.
25. Suetta, C., S.P. Magnusson, A. Rosted, et al., Resistance training in the early postoperative phase reduces hospitalization and leads to muscle hypertrophy in elderly hip surgery patients--a controlled, randomized study. J Am Geriatr Soc, 2004. **52**(12): p. 2016-22.

26. Abdellaoui, A., C. Prefaut, F. Gouzi, et al., Skeletal muscle effects of electrostimulation after COPD exacerbation: a pilot study. Eur Respir J, 2011. **38**(4): p. 781-8.
27. Maddocks, M., C.M. Nolan, W.D. Man, et al., Neuromuscular electrical stimulation to improve exercise capacity in patients with severe COPD: a randomised double-blind, placebo-controlled trial. Lancet Respir Med, 2016. **4**(1): p. 27-36.
28. Nuhr, M.J., D. Pette, R. Berger, et al., Beneficial effects of chronic low-frequency stimulation of thigh muscles in patients with advanced chronic heart failure. Eur Heart J, 2004. **25**(2): p. 136-43.
29. Vieira, P.J., A.M. Chiappa, G. Cipriano, Jr., et al., Neuromuscular electrical stimulation improves clinical and physiological function in COPD patients. Respir Med, 2014. **108**(4): p. 609-20.
30. Sillen, M.J., F.M. Franssen, J.M. Delbressine, et al., Efficacy of lower-limb muscle training modalities in severely dyspnoeic individuals with COPD and quadriceps muscle weakness: results from the DICES trial. Thorax, 2014. **69**(6): p. 525-31.
31. Vivodtzev, I., J.L. Pepin, G. Vottero, et al., Improvement in quadriceps strength and dyspnea in daily tasks after 1 month of electrical stimulation in severely deconditioned and malnourished COPD. Chest, 2006. **129**(6): p. 1540-8.
32. Maddocks, M., M. Lewis, A. Chauhan, et al., Randomized controlled pilot study of neuromuscular electrical stimulation of the quadriceps in patients with non-small cell lung cancer. J Pain Symptom Manage, 2009. **38**(6): p. 950-6.
33. Maddocks, M., V. Halliday, A. Chauhan, et al., Neuromuscular electrical stimulation of the quadriceps in patients with non-small cell lung cancer receiving palliative chemotherapy: a randomized phase II study. PLoS One, 2013. **8**(12): p. e86059.
34. Schink, K., H.J. Herrmann, R. Schwappacher, et al., Effects of whole-body electromyostimulation combined with individualized nutritional support on body composition in patients with advanced cancer: a controlled pilot trial. BMC Cancer, 2018. **18**(1): p. 886.
35. Bewarder, M., A. Klostermann, M. Ahlgrimm, et al., Safety and feasibility of electrical muscle stimulation in patients undergoing autologous and allogeneic stem cell transplantation or intensive chemotherapy. Supportive Care in Cancer, 2018.
36. Bohannon, R.W. and R. Crouch, Minimal clinically important difference for change in 6-minute walk test distance of adults with pathology: a systematic review. J Eval Clin Pract, 2017. **23**(2): p. 377-381.
37. Purcell, A., J. Fleming, S. Bennett, et al., A multidimensional examination of correlates of fatigue during radiotherapy. Cancer, 2010. **116**(2): p. 529-37.
38. Pescatello, L.S., Arena, R., Riebe, D. Thompson, P.D., ACSM's Guidelines for Exercise Testing and Prescription, 9th ed. 2014, Baltimore, MD: Lippincott Williams & Wilkins.
39. Veterans and Agent Orange: Update 2014. Mil Med, 2017. **182**(7): p. 1619-1620.

40. Anderson, L.J., C. Yin, R. Burciaga, et al., Assessing Cachexia Acutely after Autologous Stem Cell Transplant. Cancers (Basel), 2019. **11**(9).
41. Muntaner-Mas, A., A. Martinez-Nicolas, C.J. Lavie, et al., A Systematic Review of Fitness Apps and Their Potential Clinical and Sports Utility for Objective and Remote Assessment of Cardiorespiratory Fitness. Sports Med, 2019. **49**(4): p. 587-600.
42. Capela, N.A., E.D. Lemaire, and N. Baddour, Novel algorithm for a smartphone-based 6-minute walk test application: algorithm, application development, and evaluation. J Neuroeng Rehabil, 2015. **12**: p. 19.
43. Enright, P.L. and D.L. Sherrill, Reference equations for the six-minute walk in healthy adults. Am J Respir Crit Care Med, 1998. **158**(5 Pt 1): p. 1384-7.
44. Laboratories, A.T.S.C.o.P.S.f.C.P.F., ATS statement: guidelines for the six-minute walk test. Am J Respir Crit Care Med, 2002. **166**(1): p. 111-7.
45. Hooker, S.P., A. Feeney, B. Hutto, et al., Validation of the actical activity monitor in middle aged and older adults. J Phys Act Health, 2011. **8**(3): p. 372-81.
46. Burney, B.O., T.G. Hayes, J. Smiechowska, et al., Low testosterone levels and increased inflammatory markers in patients with cancer and relationship with cachexia. J Clin Endocrinol Metab, 2012. **97**(5): p. E700-9.
47. Garcia, J.M., R.V. Boccia, C.D. Graham, et al., Anamorelin for patients with cancer cachexia: an integrated analysis of two phase 2, randomised, placebo-controlled, double-blind trials. Lancet Oncol, 2015. **16**(1): p. 108-16.
48. Bailey, C., A. Asher, S. Kim, et al., Evaluating Hand Grip Strength Prior to Hematopoietic Stem Cell Transplantation as a Predictor of Patient Outcomes. Rehabilitation Oncology, 2018. **36**(3): p. 172-179.
49. Whitney, S.L., D.M. Wrisley, G.F. Marchetti, et al., Clinical measurement of sit-to-stand performance in people with balance disorders: validity of data for the Five-Times-Sit-to-Stand Test. Phys Ther, 2005. **85**(10): p. 1034-45.
50. Bruera, E., N. Kuehn, M.J. Miller, et al., The Edmonton Symptom Assessment System (ESAS): a simple method for the assessment of palliative care patients. J Palliat Care, 1991. **7**(2): p. 6-9.
51. Garcia, J.M., J. Friend, and S. Allen, Therapeutic potential of anamorelin, a novel, oral ghrelin mimetic, in patients with cancer-related cachexia: a multicenter, randomized, doubleblind, crossover, pilot study. Supportive Care in Cancer, 2013. **21**(1): p. 129-37.
52. Garcia, J.M., M. Garcia-Touza, R.A. Hijazi, et al., Active ghrelin levels and active to total ghrelin ratio in cancer-induced cachexia. J Clin Endocrinol Metab, 2005. **90**(5): p. 2920-6.
53. Lerner, L., T.G. Hayes, N. Tao, et al., Plasma growth differentiation factor 15 is associated with weight loss and mortality in cancer patients. J Cachexia Sarcopenia Muscle, 2015. **6**(4): p. 317-24.
54. Syrjala, K.L., J.C. Yi, S.B. Artherholt, et al., Measuring musculoskeletal symptoms in cancer survivors who receive hematopoietic cell transplantation. J Cancer Surviv, 2010. **4**(3): p. 225- 35.

55. Rajotte, E.J., J.C. Yi, K.S. Baker, et al., Community-based exercise program effectiveness and safety for cancer survivors. J Cancer Surviv, 2012. **6**(2): p. 219-28.
56. Smets, E.M., B. Garssen, B. Bonke, et al., The Multidimensional Fatigue Inventory (MFI) psychometric qualities of an instrument to assess fatigue. J Psychosom Res, 1995. **39**(3): p. 315-25. 57. Smets, E.M., B. Garssen, A. Cull, et al., Application of the multidimensional fatigue inventory (MFI-20) in cancer patients receiving radiotherapy. Br J Cancer, 1996. **73**(2): p. 241-5.
58. Brazier, J.E., R. Harper, N.M. Jones, et al., Validating the SF-36 health survey questionnaire: new outcome measure for primary care. BMJ, 1992. **305**(6846): p. 160-4.
59. Rennie, S., ELECTROPHYSICAL AGENTS Contraindications and Precautions: An Evidence- Based Approach to Clinical Decision Making in Physical Therapy Foreword. Physiotherapy Canada, 2010. **62**(5): p. 1-3.
