# Supplementary material for: Neuromuscular electrical stimulation for physical function maintenance during hematopoietic stem cell transplantation: Study protocol
Source: PLoS One. 2024 May 10;19(5):e0302970. doi: 10.1371/journal.pone.0302970 (PMC11086915; doi:10.1371/journal.pone.0302970)
Supplement: Attachment — Submitted filename: Response to Reviewers.docx [file pone.0302970.s003.docx]

RE: PONE-D-23-33264

Neuromuscular electrical stimulation for physical function maintenance during hematopoietic stem cell transplant: Study protocol

The authors thank the journal editorial team and the reviewers for their careful attention and thoughtful feedback which we have responded to point-by-point below. **PLEASE NOTE**: LINE NUMBERS IN AUTHOR RESPONSES REFER TO THE CLEAN MANUSCRIPT FILE.

Journal Requirements:

1. Please ensure that your manuscript meets PLOS ONE's style requirements, including those for file naming. The PLOS ONE style templates can be found at 
<https://journals.plos.org/plosone/s/file?id=wjVg/PLOSOne_formatting_sample_main_body.pdf> and <https://journals.plos.org/plosone/s/file?id=ba62/PLOSOne_formatting_sample_title_authors_affiliations.pdf>

***Response to journal comment:***

*The title page components, Abstract, all headings and reference to Figures throughout the manuscript, reducing number of abbreviations, Figure format (including upload to PACE), and file naming have been revised per guidelines provided. Please note, the main body example link provided above indicates Figure legends should be placed “in line” with the Figure label and title; however, the submission guideline site found here* [*Figures | PLOS ONE*](https://journals.plos.org/plosone/s/figures#loc-how-to-submit-figures-and-captions) *indicates the legend should be placed “after” the label and title. We have inserted the legend “after” the label and title. In addition, the title page example link provided above does not display a Short Title which is required per the submission site* [*Submission Guidelines | PLOS ONE*](https://journals.plos.org/plosone/s/submission-guidelines#loc-title) *so we have included one here.*

2. We note that the grant information you provided in the ‘Funding Information’ and ‘Financial Disclosure’ sections do not match. When you resubmit, please ensure that you provide the correct grant numbers for the awards you received for your study in the ‘Funding Information’ section.

***Please find response to journal comments #2-4 provided as one summary response after #4.***

3. Thank you for stating the following financial disclosure:  "This study was funded by a grant from the U.S. Dept. of VA (grant number RX003245)"
  
Please state what role the funders took in the study.  If the funders had no role, please state: "The funders had no role in study design, data collection and analysis, decision to publish, or preparation of the manuscript." If this statement is not correct you must amend it as needed. Please include this amended Role of Funder statement in your cover letter; we will change the online submission form on your behalf.

***Please find response to journal comments #2-4 provided as one summary response after #4.***

4. Thank you for stating the following in the Acknowledgments Section of your manuscript: 
'This study was supported by resources and facilities at the VAPSHCS Geriatric Research, Education and Clinical Center and JMG receives research support from the VA (BX002807), the Congressionally Directed Medical Research Program (PC170059), and the National Institutes of Health (NIH; R01CA239208, R01AG061558). LJA receives research support from the University of Washington (DK007247) and the VA (RX003245). RS Medical contributed the RS-4i Plus Sequential Stimulators, were involved in design of the NMES and sham parameters, and provided technical support throughout the study."

We note that you have provided funding information that is not currently declared in your Funding Statement. However, funding information should not appear in the Acknowledgments section or other areas of your manuscript. We will only publish funding information present in the Funding Statement section of the online submission form. Please remove any funding-related text from the manuscript and let us know how you would like to update your Funding Statement. Currently, your Funding Statement reads as follows: "This study was funded by a grant from the U.S. Dept. of VA (grant number RX003245)"

Please include your amended statements within your cover letter; we will change the online submission form on your behalf.

***Response to journal comments #2-4:***

*Comments #2-4 all refer to inconsistency/inaccuracy in the funding, disclosure, and/or acknowledgement sections; all three revised statements are provided below and in the revised Cover Letter. We are responding to all three here for efficiency.*

*Direct funding for the research has been removed from Acknowledgements and added to the funding statement. No financial support was received specifically for this research from the sources listed in the current Acknowledgments statement.*

*The Protocol Article Template found here indicates there should be a funding statement and a competing interest statement on the title page, yet these are not displayed on the sample title page provided in #1 above, and #4 above states you only publish funding information entered into the online submission form.* [*https://journals.plos.org/plosone/s/file?id=c9fb/Study%20Protocol%20Article%20Template.pdf*](https://journals.plos.org/plosone/s/file?id=c9fb/Study%20Protocol%20Article%20Template.pdf)

*Please clarify whether the funding and/or competing interest statements are required on the title page.*

***Funding****: This study was funded by* *a grant from the U.S. Dept. of VA (grant number RX003245; PI: Anderson). JMG receives research support from the VA (BX002807), the Congressionally Directed Medical Research Program (PC170059), and the National Institutes of Health (NIH; R01CA239208, R01AG061558).* *These funders had no role in study design, data collection and analysis, decision to publish, or preparation of the manuscript.*

***Competing interests****: The authors declare no conflicts of interest*

***Acknowledgments****: This study was supported by resources and facilities at the VAPSHCS Geriatric Research, Education and Clinical Center and JMG receives research support from the VA (BX002807), the Congressionally Directed Medical Research Program (PC170059), and the National Institutes of Health (NIH; R01CA239208, R01AG061558).* *These supporters had no role in study design, data collection and analysis, decision to publish, or preparation of the manuscript.*

5. We note that you have indicated that data from this study are available upon request. PLOS only allows data to be available upon request if there are legal or ethical restrictions on sharing data publicly. For more information on unacceptable data access restrictions, please see <http://journals.plos.org/plosone/s/data-availability#loc-unacceptable-data-access-restrictions>.

In your revised cover letter, please address the following prompts:

a) If there are ethical or legal restrictions on sharing a de-identified data set, please explain them in detail (e.g., data contain potentially sensitive information, data are owned by a third-party organization, etc.) and who has imposed them (e.g., an ethics committee). Please also provide contact information for a data access committee, ethics committee, or other institutional body to which data requests may be sent.

b) If there are no restrictions, please upload the minimal anonymized data set necessary to replicate your study findings as either Supporting Information files or to a stable, public repository and provide us with the relevant URLs, DOIs, or accession numbers. For a list of acceptable repositories, please see <http://journals.plos.org/plosone/s/data-availability#loc-recommended-repositories>.

We will update your Data Availability statement on your behalf to reflect the information you provide.

***Response to journal comment:***

*We apologize for the confusion on this. The original data statement pertained to data generated from the actual trial, it did not pertain to this submission of the study protocol. The revised statement shown here is also contained in the revised Cover Letter:*

***Data availability****: the data availability policy is not applicable as this study protocol does not report data.*

6. We note that the original protocol file you uploaded contains a confidentiality notice indicating that the protocol may not be shared publicly or be published. Please note, however, that the PLOS Editorial Policy requires that the original protocol be published alongside your manuscript in the event of acceptance. Please note that should your paper be accepted, all content including the protocol will be published under the Creative Commons Attribution (CC BY) 4.0 license, which means that it will be freely available online, and any third party is permitted to access, download, copy, distribute, and use these materials in any way, even commercially, with proper attribution.

Therefore, we ask that you please seek permission from the study sponsor or body imposing the restriction on sharing this document to publish this protocol under CC BY 4.0 if your work is accepted. We kindly ask that you upload a formal statement signed by an institutional representative clarifying whether you will be able to comply with this policy. Additionally, please upload a clean copy of the protocol with the confidentiality notice (and any copyrighted institutional logos or signatures) removed.

***Response to journal comment:***

*We thank the journal for their response to our email inquiry asking for further guidance. Regarding copyrighted logos or signatures, thank you for verifying the following “*It does appear your protocol is *already free of such incompatible elements.” Regarding the Confidentiality Notice, I can confirm that Dr. Patricia Dorn, Director of the VA Rehabilitation R&D service line (study sponsor), has determined that there are no restrictions on publication of the protocol document. I have uploaded an email from Erin Spaniol, Deputy Director of Rehabilitation R&D indicating the following statement:*

*“*We spoke with Dr. Dorn and she is in agreement that the protocol document is your intellectual property and you are able to share at your discretion.*”*

*Upon further clarification from the sponsor, we have also uploaded the consent form with name and phone number of study coordinator redacted. In addition, once enrolment is closed for this active trial, the consent form approved at that time will be published on this trial’s webpage on clinicaltrial.gov as required per VHA Directive 1200.05(3).*

[Note: HTML markup is below. Please do not edit.]

Reviewers' comments:

Reviewer's Responses to Questions

**Comments to the Author**

1. Does the manuscript provide a valid rationale for the proposed study, with clearly identified and justified research questions?

The research question outlined is expected to address a valid academic problem or topic and contribute to the base of knowledge in the field.

Reviewer #1: Yes

Reviewer #2: Yes

2. Is the protocol technically sound and planned in a manner that will lead to a meaningful outcome and allow testing the stated hypotheses?

The manuscript should describe the methods in sufficient detail to prevent undisclosed flexibility in the experimental procedure or analysis pipeline, including sufficient outcome-neutral conditions (e.g. necessary controls, absence of floor or ceiling effects) to test the proposed hypotheses and a statistical power analysis where applicable. As there may be aspects of the methodology and analysis which can only be refined once the work is undertaken, authors should outline potential assumptions and explicitly describe what aspects of the proposed analyses, if any, are exploratory.

Reviewer #1: Partly

Reviewer #2: Yes

3. Is the methodology feasible and described in sufficient detail to allow the work to be replicable?

Descriptions of methods and materials in the protocol should be reported in sufficient detail for another researcher to reproduce all experiments and analyses. The protocol should describe the appropriate controls, sample size calculations, and replication needed to ensure that the data are robust and reproducible.

Reviewer #1: Yes

Reviewer #2: Yes

4. Have the authors described where all data underlying the findings will be made available when the study is complete?

The [PLOS Data policy](https://gcc02.safelinks.protection.outlook.com/?url=https%3A%2F%2Fjournals.plos.org%2Fplosone%2Fs%2Fmaterials-and-software-sharing&data=05%7C02%7C%7C0454521844f148b9a5ff08dbffcb7080%7Ce95f1b23abaf45ee821db7ab251ab3bf%7C0%7C0%7C638385021706399562%7CUnknown%7CTWFpbGZsb3d8eyJWIjoiMC4wLjAwMDAiLCJQIjoiV2luMzIiLCJBTiI6Ik1haWwiLCJXVCI6Mn0%3D%7C3000%7C%7C%7C&sdata=shVVV4Ze6I4FmvSJl%2BWe0vUBkPpPCXc8B7UgXtSH6jQ%3D&reserved=0) requires authors to make all data underlying the findings described in their manuscript fully available without restriction, with rare exception, at the time of publication. The data should be provided as part of the manuscript or its supporting information, or deposited to a public repository. For example, in addition to summary statistics, the data points behind means, medians and variance measures should be available. If there are restrictions on publicly sharing data—e.g. participant privacy or use of data from a third party—those must be specified.

Reviewer #1: Yes

Reviewer #2: Yes

5. Is the manuscript presented in an intelligible fashion and written in standard English?

PLOS ONE does not copyedit accepted manuscripts, so the language in submitted articles must be clear, correct, and unambiguous. Any typographical or grammatical errors should be corrected at revision, so please note any specific errors here.

Reviewer #1: Yes

Reviewer #2: Yes

**PLEASE NOTE**: LINE NUMBERS IN AUTHOR RESPONSES REFER TO THE CLEAN MANUSCRIPT FILE

6. Review Comments to the Author

**Reviewer #1**: In this study protocol, the authors investigated the efficacy of NMES for mitigating the detrimental effect of hematopoietic stem cell transplant in patients with cancer. The application of NMES is patients with cancer is emerging and with a promising evidence. Therefore, new studies are welcome to have a comprehensive understanding of the efficacy and drawbacks of this strategy. I have included some comments for authors’ consideration as follows:

1. Line 71-74; The sentence needs correction.

***Response to reviewer comment:***

*We thank the reviewer for pointing out that these sentences require clarity. We have revised these lines accordingly, in combination with recommendation #2 below (now lines 66-69), which now state:*

*“For example, in a cohort of 201 HCT patients, only 2% were referred for physical therapy during the in-patient post-HCT recovery period (12). In addition, implementation of supervised exercise, like traditional resistance exercise, can be especially problematic for HCT patients who are immunocompromised and experience high levels of fatigue.”*

2. Line 66-79 (Paragraph 2); In order to shorten the introduction, the information provided in the second paragraph could be summarized. Moreover, the structure and the information provided in the second paragraph of the introduction are as if exercise is the main intervention in this study while the authors just want to justify the use of NMES instead of exercise training. So, I recommend to re-structure the second paragraph and remove unnecessary information.

***Response to reviewer comment:***

*The authors appreciate the suggestion to summarize Introduction paragraph 2 to avoid distracting the reader. We have now revised this paragraph to by reducing the length while more concisely conveying our intended message, which was correctly interpreted by this reviewer. We have also made edits throughout the Introduction to be more concise while highlighting the main innovations of the current design, as suggested below in comment #3.*

3. In the introduction, the authors have appropriately presented the current knowledge and understanding concerning the application of NMES in clinical populations such as cancer, however, it would be more interesting if the authors briefly highlight which specific gaps in the literature are covered in this study and what is the main novelty of this project.

***Response to reviewer comment:***

*We thank the reviewer for pointing out that the gaps in the literature are not obvious and should be detailed further. The following statement can now be found prior to the study goals (lines 100-104):*

*“While NMES may be safe and well-tolerated during HCT, its potential to benefit physical function and fatigue during HCT has yet to be tested in a randomized controlled setting.* *It is also unknown whether stimulating multiple lower body muscle groups and reducing prescription to three days/week to allow muscle recovery between sessions would induce a clinically meaningful impact on physical function during HCT.”*

4. I congratulate the authors for providing the detailed information in the “Participants and study design” section.

***Response to reviewer comment:*** *The authors thank the reviewer for this generous comment.*

5. Line 173; No information regarding the randomization and blinding procedure have been provided. How did the author evaluate the efficacy of the blinding strategy used in this study?

***Response to reviewer comment:***

*The reviewer brings up an important point regarding missing details about randomization and blinding. We have now clarified the method for sequence generation and stratification parameters and justified the unblind status of study coordinators (line 168-173). In addition, we have now included the following information regarding our attempt to minimize patient unblinding (lines 174-179):*

“*Participants are informed that two different electric signals are being tested in this study: one will be actively felt throughout each session, and one will only be actively felt for the first two minutes of each. This strategy was chosen to remain transparent about the intervention arms while minimizing likelihood of patient unblinding, which would be extremely high if informed of* *an ‘active’ and a ‘sham’ group. However, subjects are also informed that we do not expect every outcome to be affected in both groups, and that the primary study goal is to determine* *which outcomes are affected, if any, in each group.*”

6. Line 174; Since the replication of a study protocol by other researchers is important, I recommend the authors to provide more information regarding the “stratification by diagnosis”.

***Response to reviewer comment:***

*We thank the reviewer for pointing out this need for clarification. Most often, and certainly at our institution, the most common indication for autologous stem cell transplant is multiple myeloma. Study groups should have equal distribution of* *multiple myeloma and non-multiple myeloma (all other diagnoses). We have now specified this stratification information in line 168-170.*

7. Could the authors elaborate more on their choice of the NMES parameters? Considering the fact that the main outcome measure of this study is 6MWT and that low frequency-NMES is more likely to induce aerobic adaptations while high frequency-NMES is proven to have strengthening effect on target muscles.

***Response to reviewer comment:***

*We agree that intervention goals and outcome selection should be thoughtfully paired. We also acknowledge the appearance of discrepancy with a high frequency-NMES intervention and a primary outcome (**6MWT) that is conventionally used as a sub-maximal endurance test. High frequency-NMES (≥35Hz) has repeatedly improved 6MWT performance, used as an indicator of global physical function, in patients with chronic disease including those with advanced cancer. The 6MWT, which has established clinically meaningful criteria, also provides a means for addressing the clinical relevance of NMES in this treatment setting. We have edited and clarified the text to explain this (lines 228-231):*

*“Despite that the 6MWT is conventionally used as a sub-maximal endurance test, high frequency-NMES, such as that utilized here, has repeatedly improved 6MWT performance, and therefore is used as an indicator of global physical function, in chronic disease cohorts including those with advanced cancer [23, 30, 35-37].”*

- *[23] Maddocks M, et al. (2016). Neuromuscular electrical stimulation to improve exercise capacity in patients with severe COPD: A randomized double blind, placebo-controlled trial. The Lancet Respiratory Medicine, 4(1), 27-36.*
- *[30] Schink K, et al., (2018) Effects of whole-body electromyostimulation combined with individualized nutritional support on body composition in patients with advanced cancer: a controlled pilot trial. BMC Cancer, 18(1): 886.*
- *[35] Forestieri P, et al. (2018). Neuromuscular electrical stimulation improves exercise tolerance in patients with advanced heart failure on continuous intravenous inotropic support use-randomized controlled trial. Clin Rehabil; 32: 66–74.*
- *[36] Sumin AN, et al. (2023). Prehabilitation in Cardiovascular Surgery: The Effect of Neuromuscular Electrical Stimulation.* Int. J. Environ. Res. Public Health; 20*(3), 2678.*
- *[37] Sumin AN, et al. (2020). Neuromuscular electrical stimulation in early rehabilitation of patients with postoperative complications after cardiovascular surgery: a randomized controlled trial. Medicine; 99: e22769.*

8. Line 255, Please provide a reference for using one-minute rest between MVICs.

***Response to reviewer comment:***

*We refer the reviewer to Miyahara et al. [Effects of Proprioceptive Neuromuscular Facilitation Stretching and Static Stretching on Maximal Voluntary Contraction. Journal of Strength and Conditioning Research; 27(1): 195-201] who tested 3 MVIC repetitions of 4-seconds duration with 1-minute rest in between. Those authors reported an intraclass correlation coefficient of 0.87 (95% CI; 0.62-0.96) for the 3 serial measurements and a test-retest Pearson correlation of 0.92. This reference is now added to the manuscript with the MVIC description (line 274, Ref #42).*

9. Line 261; Instead of providing justifications for the choice of instruments used for “Patient-Reported Outcomes”, I recommend the authors to provide detailed information regarding the scoring and interpretation of these measures.

***Response to reviewer comment:***

*We thank the reviewer for making this suggestion and we agree that it will be very useful for readers to have this information, and for this level of detail to be documented here for our ability to reference in the future. This information has now been added throughout the “Patient reported outcomes” section (lines 282-323). We also feel that it remains important to show some level of justification for our assessment choice, so we have not removed that information.*

10. Line 304; Please provide more information regarding the incremental test on the ergometer for measuring peak aerobic capacity. What was the initial workload? How much load was added at each stage and what was the duration of each stage? Was the verbal encouragement provided? When the participants reported their RPE and by which Borg scale?

***Response to reviewer comment:***

*The authors appreciate the reviewer’s attention to detail on this complicated assessment. None of the procedures listed under the section “Outcomes temporarily on hold due to COVID-19 precautions” have been performed in the current study to date due to COVID precautions remaining in place by the Bone Marrow Transplant Unit. However, our laboratory routinely performs aerobic capacity tests for our other clinical studies and provides frequent verbal encouragement throughout each test. We obtain RPE during the cooldown period, once the mask can be removed, using the Borg scale ranging from 6-20 points, as referenced here [#59: Borg GA. Psychophysical bases of perceived exertion. MSSE; 1982]. The aerobic test* *is a ramping protocol starting from 0 watts with a continuously increasing workload of 15, 20, or 25 watts/minute as determined by the calorimeter’s software estimation of the individual’s aerobic capacity, using age and body weight. Due to the unforeseen circumstance of COVID-19, aerobic capacity is not likely to be assessed on any participant in this study. We have changed the title of this section to “Outcomes proposed but unlikely to be assessed due to COVID-19 precautions” and provided these requested test details in lines 360-368.*

11. Line 402; Did the authors have any control on nutritional status of the participants during the project (in particular between FU1 and FU2)? If so, please provide the related information, if not, it could be another limitation of the study.

***Response to reviewer comment:***

*The authors thank the reviewer for this insightful comment. There are only three Bone Marrow Transplant Units in the Veteran Affairs system, and as a result, most participants return to their home state directly after Follow-Up 1. We have now added the following sentence to the limitations (lines 475-477):*

*“In addition, the lack of ability to control for nutrition, particularly during the post-intervention period, may contribute to* *physical function and quality of life at FU2, and could be an important factor to be addressed in future studies.”*

12. Line 403-407; I addressed this issue in my 5th comment. I think it is necessary that the author provide more information regarding any strategies used for blinding in the related section of the manuscript. Nevertheless, I agree that the nature of NMES makes it difficult to blind the participants.

***Response to reviewer comment:***

*We thank the reviewer for this acknowledgement, and we agree that further details should be provided. Please refer to our response in your 5^th^ comment for this information (lines 168-173).*

**Reviewer #2**: As the statistical reviewer I will focus on methods and reporting. This is a well planned and reported study. However, I have some comments to make.

1) the term pilot is used at times. As far as I understand, this relates to very small trials that obtain an effect estimate to be used for a power calculations towards designing a larger trial. Some clarification is needed, as to whether that is the case here, or the term trial needs to be dropped.

***Response to reviewer comment:***

*We appreciate this perspective and agree that the term pilot should be dropped here to avoid confusion. We have now removed that term in line 105.*

2) the authors estimate an attrition rate of 50% and they need 30 participants, but they aim to recruit 46 rather than 60 - is there a mistake somewhere?

***Response to reviewer comment:***

*Thank you for pointing out this typo, which was carried over from the expectation of NMES attenuating a sham 6MWT change by 50%. What we meant to state was that we expect a 33% attrition rate, which would result in needing 23/group (46 total). This correction has now been made (line 398).*

3) power may be inadequate for other outcomes, so this needs to be discussed as a limitation and needs to be reported when the authors conduct their analyses.

***Response to reviewer comment:***

*The authors thank the reviewer for this important consideration, and have now added the following statement to the limitations (lines 477-478):*

*“Also, this trial was only statistically powered for the primary outcome and may be under-powered for remaining outcomes, which will need to be considered during final data analysis.”*

4) randomisation may not work that well for a sample this small, so maybe the authors could consider more deterministic approaches, for example please see <https://www.jstatsoft.org/article/view/v055c01>. They may want to ignore this advice, but this would be my preferred approach. Randomisation may work fine, by chance.

***Response to reviewer comment:***

*The authors greatly appreciate this insightful feedback. We agree this suggested approach would be very valuable and we are glad to now have this resource to aid future study design. However, subject recruitment, and therefore randomization, is already underway for this trial so we will have to maintain the current randomization procedures for consistency. Although we have now added the following statement to the limitations section in acknowledgement of this point (lines 478-480):*

*“Finally, external validation will be required for any observations reported from these proposed analyses to ensure confidence in generalizability to the broader patient population.”*

5) and regression models will not work well with 30 subjects, so you may want to control for numerous covariates where imbalances exist, but in practice you will struggle to include more than a handful of covariates, especially if categorical. So it's better to strive for balance, for at least a few key covariates using a deterministic algorithm in my view, although the size of the sample means some imbalances will definitely exist.

***Response to reviewer comment:***

*We agree with the reviewer that it would not be realistic to expect more than 2-3 covariates to factor into a regression model here, especially if any of the covariates are categorical. We expect that randomization, with stratification to ensure equal distribution of gender (male vs. female) and diagnosis (multiple myeloma vs. other diagnoses), now clarified in lines 168-173. We will test that other factors have equal distribution between groups for relevant biological factors such as race, ethnicity, body composition, and functional status. For consistency with the Expected Outcomes section, we have now specified that linear regression is part of the exploratory analysis strategy (line 411-413).*

7. PLOS authors have the option to publish the peer review history of their article ([what does this mean?](https://gcc02.safelinks.protection.outlook.com/?url=https%3A%2F%2Fjournals.plos.org%2Fplosone%2Fs%2Feditorial-and-peer-review-process%23loc-peer-review-history&data=05%7C02%7C%7C0454521844f148b9a5ff08dbffcb7080%7Ce95f1b23abaf45ee821db7ab251ab3bf%7C0%7C0%7C638385021706399562%7CUnknown%7CTWFpbGZsb3d8eyJWIjoiMC4wLjAwMDAiLCJQIjoiV2luMzIiLCJBTiI6Ik1haWwiLCJXVCI6Mn0%3D%7C3000%7C%7C%7C&sdata=USQRTdB7NbHPT%2B0uVCg828P0sSC%2B7g7sFU4Tx51Qyr0%3D&reserved=0)). If published, this will include your full peer review and any attached files.

If you choose “no”, your identity will remain anonymous but your review may still be made public.

**Do you want your identity to be public for this peer review?** For information about this choice, including consent withdrawal, please see our [Privacy Policy](https://gcc02.safelinks.protection.outlook.com/?url=https%3A%2F%2Fwww.plos.org%2Fprivacy-policy&data=05%7C02%7C%7C0454521844f148b9a5ff08dbffcb7080%7Ce95f1b23abaf45ee821db7ab251ab3bf%7C0%7C0%7C638385021706399562%7CUnknown%7CTWFpbGZsb3d8eyJWIjoiMC4wLjAwMDAiLCJQIjoiV2luMzIiLCJBTiI6Ik1haWwiLCJXVCI6Mn0%3D%7C3000%7C%7C%7C&sdata=13gdTW2kbShw3ojEg6NyjZEda8Wm4IWs4Mo3UqaVzWo%3D&reserved=0).

Reviewer #1: **Yes:** Ehsan Amiri

Reviewer #2: No
